# Cassava Starch Films Containing Quinoa Starch Nanocrystals: Physical and Surface Properties

**DOI:** 10.3390/foods12030576

**Published:** 2023-01-28

**Authors:** Lía Ethel Velásquez-Castillo, Mariani Agostinetto Leite, Victor Jesús Aredo Tisnado, Cynthia Ditchfield, Paulo José do Amaral Sobral, Izabel Cristina Freitas Moraes

**Affiliations:** 1 Postgraduate Program in Materials Science and Engineering, University of São Paulo, USP/FZEA, Av. Duque de Caxias Norte, 225, Pirassununga 13635-900, SP, Brazil; 2 Department of Food Engineering, Faculty of Animal Science and Food Engineering, University of São Paulo, Pirassununga 13635-900, SP, Brazil; 3 Food Research Center (FoRC), University of São Paulo, Rua do Lago, 250, Semi-Industrial Building, Block C, São Paulo 05508-080, SP, Brazil

**Keywords:** *Chenopodium quinoa* Willd., starch, nanocrystal, solution casting, nanocomposite films

## Abstract

Quinoa starch nanocrystals (QSNCs), obtained by acid hydrolysis, were used as a reinforcing filler in cassava starch films. The influence of QSNC concentrations (0, 2.5, 5.0, 7.5 and 10%, *w*/*w*) on the film’s physical and surface properties was investigated. QSNCs exhibited conical and parallelepiped shapes. An increase of the QSNC concentration, from 0 to 5%, improved the film’s tensile strength from 6.5 to 16.5 MPa, but at 7.5%, it decreased to 11.85 MPa. Adequate exfoliation of QSNCs in the starch matrix also decreased the water vapor permeability (~17%) up to a 5% concentration. At 5.0% and 7.5% concentrations, the films increased in roughness, water contact angle, and opacity, whereas the brightness decreased. Furthermore, at these concentrations, the film’s hydrophilic nature changed (water contact angle values of >65°). The SNC addition increased the film opacity without causing major changes in color. Other film properties, such as thickness, moisture content and solubility, were not affected by the QSNC concentration. The DSC (differential scanning calorimetry) results indicated that greater QSNC concentrations increased the second glass transition temperature (related to the biopolymer-rich phase) and the melting enthalpy. However, the film’s thermal stability was not altered by the QSNC addition. These findings contribute to overcoming the starch-based films’ limitations through the development of nanocomposite materials for future food packaging applications.

## 1. Introduction

Currently, the development and applications of biopolymer-based biodegradable films are one of the main interesting trends in food and materials sciences for the replacement of synthetic polymers, which are derived from the petrochemicals used in packaging [1,2]. Among biopolymers, starches are a good alternative to producing these materials because they are biodegradable, from natural and renewable sources and are produced throughout the world at a relatively low cost. Indeed, there are several conventional and unconventional starch sources. Within the conventional varieties, Cassava (*Manihot esculenta*) is an important source of relatively cheap starch, possessing excellent film-forming properties [3,4].

Starch-based films present desirable characteristics for food packaging applications because they are homogeneous, odorless, tasteless, colorless, and non-toxic, and are semipermeable to oxygen, carbon dioxide and flavor components [5]. Despite these benefits, starch-based films still have some disadvantages when compared to conventional plastics, such as their hydrophilic character, weak mechanical properties and high-water vapor permeability, which have limited their potential for industrial applications [6]. An alternative for enhancing this material is by using nanomaterial filler as a reinforcement, considering that bio-based nanocomposite films are safe, non-toxic, and a “greener” option [7]. Specifically, nanomaterials produced from starch have been applied as a reinforcing filler in starch-based films, improving their mechanical and barrier properties [8,9,10].

The interest in this type of nanomaterial is growing because of their abundance, biodegradability, biocompatibility and particularly for coming from a renewable resource [11]. The most common nanoparticles from this source are starch nanocrystals (SNCs) which have become a suitable alternative because of their reduced cost and straightforward production method [12]. SNCs are crystalline residues obtained by acid hydrolysis of the starch granule’s amorphous area, degraded below the gelatinization temperature [13,14].

Several authors observed that the addition of SNCs from different sources, such as waxy maize, rice, taro and potato, into starch (also from different sources)-film matrices improved the films’ mechanical properties [15,16,17], and in some cases, also improved the water vapor permeability and thermal properties of the films [2,10,18,19]. These improvements were caused by strong interactions between SNCs and starch molecules and their compatibility with the starch matrices. Nevertheless, starch characteristics (yield and morphology) and SNC processing conditions (concentration, pH, and compatibility with the starch matrices) can influence the effect of SNCs on material properties, while the SNC botanical source can define its structural and morphological properties.

In this sense, the A-type starches have been associated with SNCs with platelet morphology and greater thermal stability [20], which is advantageous for nanocomposite applications because of their potential to increase the film barrier and reduce thermal degradation. Furthermore, starches with small granules and low amylose contents tend to facilitate acid hydrolysis, resulting in a short hydrolysis period [21,22]. An interesting starch source that presents these characteristics is Quinoa (*Chenopodium quinoa* Willd.). Quinoa is an Andean grain crop with 53.5–69.2% of its dry weight as starch, its main storage compound [23], making it an attractive, unconventional source for starch isolation.

Quinoa starch (QS) presents with an A-type crystalline pattern, a small granule size (0.5–3 μm) with polygonal shapes, and low amylose content (7–26%) and gelatinization temperatures (54–78 °C) [23], which are interesting characteristics for SNC production. In previous work, Velásquez-Castillo et al. [14] produced SNCs from QS (QSNC) with suitable properties to be used in starch-based films as a reinforcing filler.

Currently, there is limited research on the application of SNCs from unconventional sources as a reinforcing filler in starch-based films; although potato, rice and taro starches have been studied [2,17,19], the vast majority focused on SNCs obtained from waxy maize [10,18,24,25]. In this regard, the use of QSNC, an A-type crystalline pattern starch with excellent properties for SNC production, for the development of nanocomposite films contributes to this field. Moreover, although several authors have discussed the effects of SNC additions on the mechanical and barrier properties of starch films, other important properties, such as starch films’ solubility in water [2,19] and surface properties (the roughness and contact angle), are presented with limited information [25]. Therefore, the main objectives of this study were to develop films based on cassava starch (CS) containing QSNC and to evaluate the effect of its concentration on the physical and surface properties of the material. The main hypothesis is that QSNC would reinforce the CS film, thus improving its properties.

## 2. Materials and Methods

### 2.1. Materials

Quinoa grains of the “Real” variety originally from Bolivia were bought in Pirassununga (Sao Paulo, Brazil). Commercial cassava starch (CS) was acquired from H. L. do Brasil Indústria e Comercio de Produtos Alimentícios LTDA in São Paulo, Brazil (latitude 21°59′46″ S, longitude 47°25′33″ W). The cassava starch contained 12.31 ± 0.04% moisture content (w.b.), 0.39 ± 0.02% lipids (d.b.), 0.16 ± 0.00% ash (d.b.), 0.26 ± 0.02% protein (d.b.), and 16.90 ± 0.18% (starch basis) apparent amylose content; these results were obtained by employing the AOAC International standard methods [26] and the International Organization for Standardization (ISO) method 6647:1987 [27].

Quinoa’s starch extraction yield, chemical composition and apparent amylose content have been previously reported in detail by Velásquez-Castillo et al. [14]. The reagents were purchased from Quimica Moderna, São Paulo, Brazil (Sulfuric acid, 95–98%); Sigma-Aldrich Corporation (potato amylose-A0512 and amylopectin-A8515, used in the determination of the apparent amylose content); Labsynth company, Sao Paulo, Brazil (glycerol, citric acid, iodine, potassium iodide, glacial acetic acid, sodium hydroxide, ethanol, petroleum ether, potassium and copper sulfate, boric acid and potassium chloride); and Merck Millipore corporation (hydrochloric acid).

### 2.2. Starch Isolation and Nanoparticles Production

#### 2.2.1. Quinoa Starch Isolation

The QS was isolated by an alkaline method, as detailed by Velásquez-Castillo et al. [14]. Briefly, quinoa grains were washed, steeped, and milled with distilled water. The resultant slurry was filtered and decanted for 12 h at 4 °C. The precipitate (which contains starch) was centrifuged for 10 min at 3830 rpm, then the ochre upper layer was removed, and the starch was resuspended in distilled water and dried for 24 h at 30 °C. The obtained starch was purified by suspending it in 0.3% NaOH (*w*/*w*), neutralized with a citric acid solution (1 M), washed with distilled water and dried. The isolated QS was ground, then sieved through a 100-mesh size and kept at room temperature.

#### 2.2.2. Quinoa Starch Nanocrystal Production

The QSNCs were prepared by acid hydrolysis of QS [14,28]. The QS (36.5 g) was mixed with 250 mL of a 3.16 M H_2_SO_4_ solution. The resulting suspension was incubated at 35 °C with stirring at 200 rpm for 5 days. The final suspension was washed with distilled water using successive centrifugations (Himac CR 21GII, Hitachi, Chiyoda, Tokyo, Japan) until neutrality, and then the precipitate was freeze-dried (FD 1.0–60E, Heto-Holten A/S, Allerod, Frederiksborg, Denmark) to obtain powdered QSNC.

### 2.3. CS, QS and QSNC Characterizations

#### 2.3.1. CS, QS and QSNC Morphologies

The CS and QS morphologies were analyzed by scanning electron microscopy (SEM) (TM3000, Hitachi Ltd., Tokyo, Japan) at 15 kV, and the QSNC were studied by field emission gun scanning electron microscopy (SEM-FEG) (XL-30 FEG, Philips Electron Optics B.V., Eindhoven, The Netherlands) at 10 kV, allowing magnifications of 3000× and 50,000×, respectively [14]. The QSNC sizes were measured by atomic force microscopy (AFM) (Solver Next, NT-MDT, Moscow, Russia), as detailed by Velásquez-Castillo et al. [14]. Briefly, the force atomic micrographs were subjected to a line-height profile analysis using the Gwyddion 2.50 software. Approximately 80 individual nanoparticles were analyzed to determine the diameter and thickness.

#### 2.3.2. CS, QS and QSNC X-ray Diffraction Patterns

The CS, QS and QSNC X-ray diffraction patterns were obtained employing a diffractometer (Miniflex600, Rigaku, Tokyo, Japan) with Cu Kα radiation (λ = 1.54056 Å) at 40 kV and 15 mA. Samples (30 mm × 40 mm) were analyzed from 4 to 40° (2θ) at 2°/min [19]. The crystallinity index was calculated as a ratio between the area of the peaks (crystalline portion) to the total area of the diffractogram [29] using the Origin software 2022b (Originlab Corporation, Northampton, MA, USA).

### 2.4. Film Preparation

The films were prepared by the casting technique according to Li et al. [18], with some modifications. CS dispersions at 4% (*w*/*w*) were prepared in distilled water and were then heated at 90 °C for 30 min under mechanical stirring at 300 rpm to allow starch gelatinization. The plasticizer, glycerol at 25% (*w*/*w*, glycerol/CS), was added, and the film-forming dispersions were stirred for 15 min and cooled to 50 °C. QSNC, previously dispersed in distilled water using an Ultraturrax (Ultraturrax^®^ IKA T25, Labotechnik, Staufen, Baden-Württemberg, Germany) at 10,000 rpm for 10 min, was added to the CS dispersions at 0, 2.5, 5.0, 7.5 and 10% (*w*/*w*, QSNC/CS) concentrations under stirring at 300 rpm for 15 min, producing film-forming dispersions (FFD).

The FFDs (about 48 g) were spread evenly over Petri dishes (14 cm diameter) and dried in a forced-air circulation oven (MA 035, Marconi, Brazil) at 30 °C for 21 h. The films were peeled off from the supports and conditioned in desiccators containing NaBr-saturated solutions (58% RH) at 25 °C for at least 5 days before the characterizations were made. For the microstructure and FTIR analyses, the samples were conditioned over silica gel at 25 °C for at least 7 days.

### 2.5. Film Characterizations

#### 2.5.1. Film Thickness, Moisture Content and Solubility in Water

Film thickness was measured using a digital micrometer (Absolute ID-C112B, Mitutoyo, Kamata, Tokyo, Japan) with an accuracy of 0.001 mm. Thickness values were considered as the mean of ten measurements taken at random points [6,18]. The film’s moisture content (MC) was determined using a gravimetric method [18,19]. Samples (20 mm diameter) were dried in an oven at 105 °C for 24 h, and the weight was registered before and after drying. The MC was calculated as g water/100 g of wet film.

Film solubility in water (SW) was determined gravimetrically [6]. Previously weighed samples (20 mm diameter) were immersed in distilled water (50 mL) and kept under mechanical stirring (60 rpm) at 25 °C for 24 h. The initial and final dry masses of the samples were determined by drying them in an oven at 105 °C for 24 h. SW (%) was calculated as the ratio between the initial and final dry mass difference and the initial dry mass of the CS films.

#### 2.5.2. Film Water Vapor Permeability

Film water vapor permeability (WVP) was determined with the ASTM E96/E96M method [30]. Circular samples were fixed onto aluminum permeation cells containing silica gel (0% RH) and placed into desiccators with distilled water at a constant temperature (25 °C). The cells were weighed in a semi-analytic balance (Marte, AS2000) every 24 h for 7 days. Then, WVP was calculated with Equation (1):

WVP = g.x/t.A.(P_1_ − P_2_) (1)

where g/t is the slope of the mass gain–time curve (g/h); x is the sample thickness (mm); A is the permeation area (0.0032 m^2^), P_1_ is the vapor pressure of pure water at 25 °C (3.166 kPa), and P_2_ is the vapor pressure of the atmosphere with silica gel (0 kPa).

#### 2.5.3. Film Color and Opacity

The film’s color was determined using a colorimeter (Miniscan MSEZ 1049, HunterLab, Reston, VA, USA) in the reflectance mode with a D65 illuminant, 10° angle and 30 mm aperture. The CIELab standard was used to measure the film’s luminosity (L*), which ranged from 0 (black, meaning dark color) to 100 (white, meaning light color) with a* varying from green (−a) to red (+a) and b* from blue (−b) to yellow (+b). The films were placed onto white and black standard plates (L* =94.51 ± 0.03, a* = −0.76 ± 0.01, b* = 2.07 ± 0.01), and then the total color difference (∆E*) was calculated using Equation (2).

ΔE* = [(L*_sample_ − L*_standard_)^2^ + (a*_sample_ − a*_standard_)^2^ + (b*_sample_ − b*_standard_)^2^]^1/2^ (2)

The film opacity was determined with the HunterLab method in reflectance mode, employing the same colorimeter as for the color measurement. The opacity (Y = Y_b_/Y_w_) was determined as the ratio between the film opacity when placed onto black (Y_b_) and white (Y_w_) standard plates [6].

#### 2.5.4. Film Surface Microstructure

The film’s microstructure (air-side surface) was analyzed using an atomic force microscope (Solver Next, NT-MDT, Moscow, Russia) operating in a semi-contact mode (resonance frequency: 150 kHz; contact force: 5 N/m; scanning speed: 0.4 Hz) [6]. Different samples were analyzed at random points with areas of 2500 µm^2^ and a 0.1 µm/pixel resolution. The roughness parameters, the average roughness (R_a_: average of the absolute value of the height deviations from a mean surface) and the root mean square roughness (R_q_: root mean square average of the height deviations taken from the mean data plane) were calculated using the microscope software (Image Analysis 3.2.5).

#### 2.5.5. Film Gloss

Film gloss was measured according to the ASTM D2457 standard [31] using a Glossimeter (NGL 20/60, Rhopoint, Bexhill on Sea, East Sussex, England UK). The samples were analyzed on the air-side surface at 10 random points at an angle of 60°.

#### 2.5.6. Film Water Contact Angle

The film water contact angle (WCA) was measured using an optical tensiometer (Attension Theta Lite, KSV Instruments, Helsinki, Uusimaa, Finland), as indicated in the ASTM D7334 standard method [32]. The samples (20 mm × 30 mm) were attached to the equipment support, and then a drop of Milli-Q water (5 µL) was dripped onto the air-side surface of the film with a precision syringe. The samples were photographed for 40 s. The sessile drop method at 30 s was used to obtain the contact angle with the Attension Theta software (Version 4.1.9.8).

#### 2.5.7. Film X-ray Diffraction Patterns

Film X-ray diffraction patterns were obtained as described in Section 2.3.2.

#### 2.5.8. Film Mechanical Properties

Film mechanical properties were determined by the uniaxial tensile tests according to ASTM D882-10 [33], using a texturometer (TA.XT2i, Stable Micro Systems, Godalming, Surrey, England) at 25 °C. The samples (90 mm × 15 mm) were fixed on the grips with a 50 mm initial separation, and the tests were carried out at 1.0 mm/s. The stress–strain curve was used directly to obtain the tensile strength (MPa) and elongation at break (%), while Young’s modulus (MPa) was calculated as the slope of the stress–strain curve at the linear region.

#### 2.5.9. Film Thermal Properties

Film thermal properties were analyzed using a differential scanning calorimeter (DSC T2010, TA Instruments, New Castle, DE, USA). Furthermore, 10 mg samples were placed in an aluminum capsule, hermetically sealed, and analyzed from −150 to 250 °C at 10 °C/min [6]. The glass transition temperature (T_g_), the melting temperature (T_m_), and the melting enthalpy (ΔH) were determined directly from the heat-flow curves using the Universal Analysis V1.7F software (TA Instruments).

The film’s thermal stability was analyzed using a thermogravimetric analyzer (STA 449F3, NETZSCH, Selb, Bavaria, Germany). Furthermore, 10 mg samples were placed in the sample holder and heated from 30 to 600 °C at 5 °C/min under an inert air atmosphere (20 mL/min) [18].

#### 2.5.10. Fourier Transform Infrared Spectroscopy

The film’s chemical structure was studied using a Fourier transform infrared (FTIR) spectrometer (Spectrum-One, PerkinElmer Inc., Waltham, MA, USA) with a universal attenuator total reflectance accessory. The spectral range was from 600–4000 cm^−1^; resolution: 4 cm^−1^; 32 scans per sample [3].

### 2.6. Statistical Analysis

All tests were performed in triplicate. The results were expressed as mean ± standard deviation and evaluated by the analysis of variance. The Statistica software (version 7.0; StatSoft Inc., Tulsa, OK, USA) was used to analyze the means with Tukey’s test at a 5% significance level.

## 3. Results

### 3.1. QS, CS and QSNC Characteristics

#### 3.1.1. QS, CS and QSNC Morphologies

**Figure 1:**
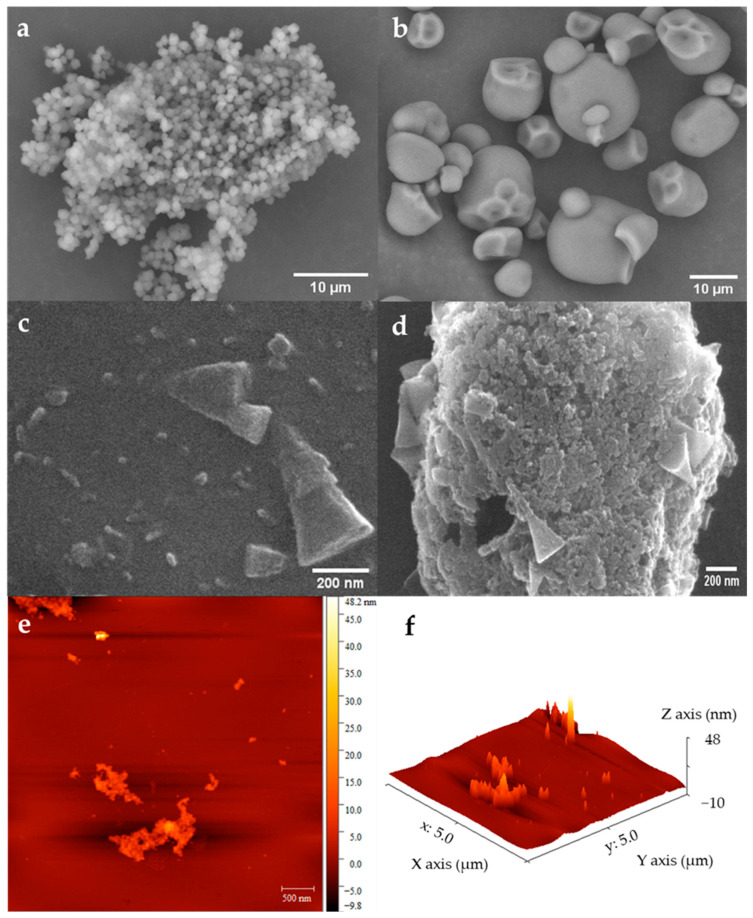
Micrographs of native starches and quinoa starch nanocrystals: (**a**) quinoa starch and (**b**) cassava starch by SEM; (**c**) quinoa starch nanocrystals and (**d**) quinoa starch nanocrystals aggregate by SEM-FEG; (**e**) 2D and (**f**) 3D quinoa starch nanocrystals by AFM.

The QS granules presented spherical shapes in a very low dimension and were disposed of in aggregates structured by a protein matrix (Figure 1a). Araujo-Farro et al. [34] isolated QS with a lower protein content of 0.9% than this study (1.6%, d.b.) and also observed a similar granule morphology. Notably, CS granules that were bigger than QS had a round shape with a smooth surface and partially inward curvatures associated with their formation and extraction processes (Figure 1b). Similar morphology has been observed by Valencia et al. [35] when analyzing a commercial CS.

Conversely, the QSNCs appeared as individual parallelepiped and conical structures, respectively (Figure 1c), which also formed micrometric-size aggregates (Figure 1d) due to the hydrogen bonding between the hydroxyl groups present on their surfaces [36]. The parallelepiped shape can be associated with amylopectin and amylose chain packing configurations in the nano-sized semi-crystalline blocklets. This result was in accordance with those observed by some authors who obtained SNCs from the A-type starches with square and parallelepiped shapes [20,21,22]. In addition, the conical structures observed in the QSNC production could be associated with a lower hydrolysis degree of the QS granules at preparing conditions.

The AFM micrographs of QSNCs (Figure 1e,f) allowed us to estimate the sizes of the parallelepiped and conical structures observed by SEM-FEG, ranging from ~50–100 nm and ~100–300 nm in the major axis (diameter) and 5–8 and 30–90 in height (thickness), respectively. These values were in accordance with the literature [14] and corroborated the nanometric scale of these nanoparticles.

#### 3.1.2. QS, CS and QSNC X-ray Diffraction Patterns

**Figure 2:**
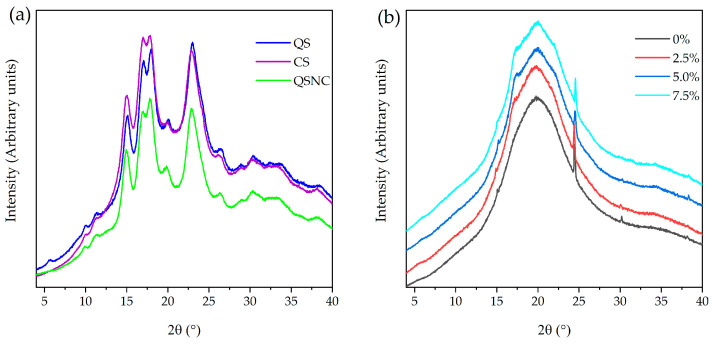
X-ray diffraction patterns: (**a**) of quinoa (QS) and cassava (CS) starches and quinoa starch nanocrystals (QSNC), and (**b**) of cassava starch films containing quinoa starch nanocrystals at different concentrations.

QS, CS and QSNC X-ray diffraction (XRD) patterns presented broad peaks with high intensity at (2θ) 15°, 17°, 18° and 23° (Figure 2a), typical of type-A crystalline starches [21,37]. These broad peaks are typical of semi-crystalline structures and are a consequence of the alternation of amorphous and crystalline layers from amylose and amylopectin that form the granule [38]. In addition, the QSNC XRD pattern suggested that QS crystallinity was preserved after acid hydrolysis in accordance with De la Concha et al. [21] and Mukurumbira et al. [22] working with this type of starch nanoparticle.

The crystallinity indices from CS, QS and QSNC were 28.6, 26.6 and 34.5%, respectively. These values were in accordance with the literature for the starches [37,39] and SNCs (35–52%) [20,21,22]. The increase observed in the QSNC crystallinity index, when compared to QS, evidenced the preferential hydrolysis of the amorphous regions of the starch granules.

### 3.2. Film Characteristics

**Figure 3:**
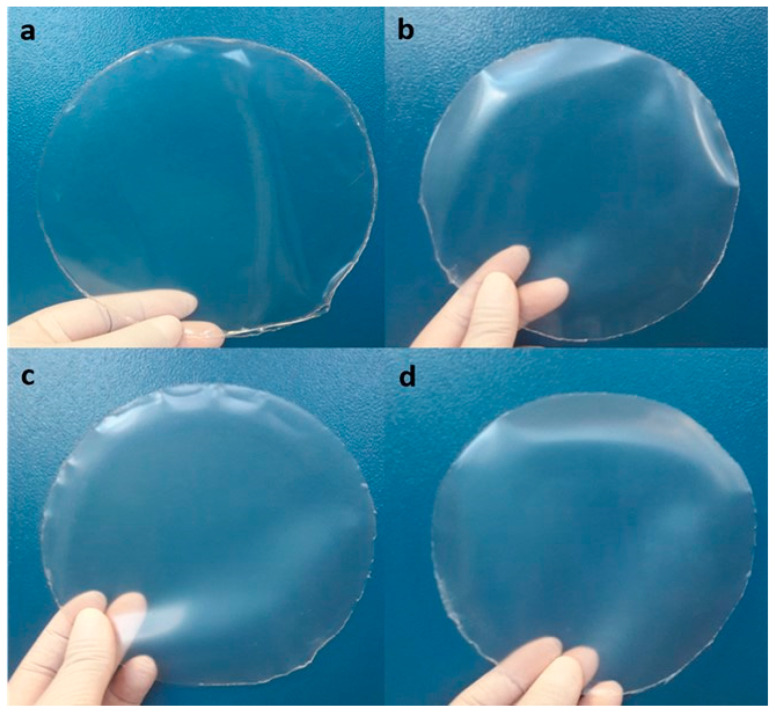
Cassava starch films containing quinoa starch nanocrystals at (*w*/*w*): (**a**) 0%, (**b**) 2.5%, (**c**) 5.0% and (**d**) 7.5%.

Films up to 7.5% of QSNC were easily handled and removed from the Petri plates and presented macroscopic homogeneity (Figure 3). The film containing 10% of QSNC cracked during drying, and thus, its characterization was not possible.

#### 3.2.1. Film Thickness, Moisture Content and Solubility in Water

The film thickness was 0.103 mm on average, with no significant changes due to the different QSNC concentrations (Table 1), indicating that this nanomaterial did not alter the sample compaction degree or film density. Similarly, Condés et al. [36] and Costa et al. [40] did not observe significant differences in the thickness with the addition of waxy and normal maize SNCs (0–12%) in amaranth protein films (~0.070 mm) or in potato, maize and cassava SNCs (0–5%) in cassava starch films (~0.100 mm), respectively. Li et al. [18] reported, however, that waxy maize SNCs (0–9%) provoked an increased thickness of pea starch films from 0.104 to 0.131 mm. However, this must have been caused by different FFD dry masses poured onto the support.

The film MC was approximately 11% and not significantly affected by the QSNC concentration (Table 1). This behavior differed from that observed by Li et al. [18], who observed a decrease in MC from 38% to 26% in pea starch films with the addition of waxy maize SNCs (0–9%). This property can vary depending on the SNC source [36] and film starch source [19], as well.

Film SW remained around 24% and was also not affected by the QSNC concentration (Table 1). Similar findings were observed for the taro and potato starch films reinforced with taro SNCs (5–10%) [19] and in amaranth protein films reinforced with waxy maize SNCs (3–9%) [36]. This behavior can be related to the QSNCs and starch matrix hydrophilic character and can also be due to the water-sensitive physical interactions between them that were disrupted by submersion in water [36]. By contrast, Jiang et al. [9] reported a decrease of SW in starch films reinforced with potato starch nanoparticles (0–9%), which were related to strong interactions from the formation of hydrogen bonding between the starch matrix and the nanoparticle.

#### 3.2.2. Film Water Vapor Permeability

Only the CS film with 5% QSNCs presented a significantly lower WVP value than the pure film (Table 1). This can be attributed to the higher concentration having a better QSNC dispersion in the film matrix. The complete exfoliation of nanoparticles can increase the film tortuosity, making water diffusion difficult [41]. At a 7.5% concentration, the WVP was not significantly different from that observed for the pure starch film, possibly due to poor exfoliation of the QSNCs that facilitated water molecule diffusion through the nanocomposite. Several authors reported that the addition of SNCs (0.1–10%) in the starch-based films decreased the WVP by 15–60% and that this property gradually decreased when the SNC concentration increased [2,19,25]. However, Li et al. [18] also reported a WVP decrease in pea starch films only at a 5% SNC concentration, as observed in this study. On the other hand, González et al. [10] did not observe changes in the WVP with the waxy maize SNC addition (0–5%) in starch films. The varying behavior observed for the WVP can be related to the SNC characteristics, such as shape, hydrophobicity, polarity, the formation of agglomerates, and crystallinity [2].

#### 3.2.3. Film Color and Opacity

Despite the significant effect in L* (Table 1), all films can be considered light in color (L* > 90). Similar behaviors were observed for a* and b*, which stayed closer to 0, so these films were colorless, as was confirmed by the low ∆E* (2.4–3.2) values (Table 1), characteristics of starch-based films [6,34,42]. This means that QSNC did not act as a pigment in the cassava starch films.

Condés et al. [36] also reported that the presence of normal and waxy maize SNC (0–12%) in amaranth protein films did not affect the color parameters. While Li et al. [18] observed that the addition of waxy maize SNC (0–9%) to pea starch films did not affect the L* and a* parameters, the films became yellow, significantly increasing the b* parameter. Dai et al. [25] also verified a significant increase in the b* parameter of cassava starch films reinforced with waxy maize cross-linked SNC (6–12%) when the concentration was above 10%, and it was attributed to the SNC aggregation.

Conversely, film opacity increased (*p* < 0.05) from 0.5 to 3.3 because of the increase of the QSNC concentration from 0 to 7.5% (Table 1). This behavior could be attributed to the reduction in light transmittance caused by the SNC distribution in the interspaces of the starch film [8]. Similar changes in opacity were observed in starch films reinforced with SNC at similar concentrations (2.5–10%) [18,19] and lower SNC concentrations (0.1–0.3%) [2]. High opacity in the films could contribute to the preservation of the food quality susceptible to light, reducing the photocatalytic reactions that contribute to food oxidation. Nevertheless, film optical transparency is a desired property with the addition of nanoparticles and is an indication of good dispersion in the film.

#### 3.2.4. Film Surface Microstructure

**Figure 4:**
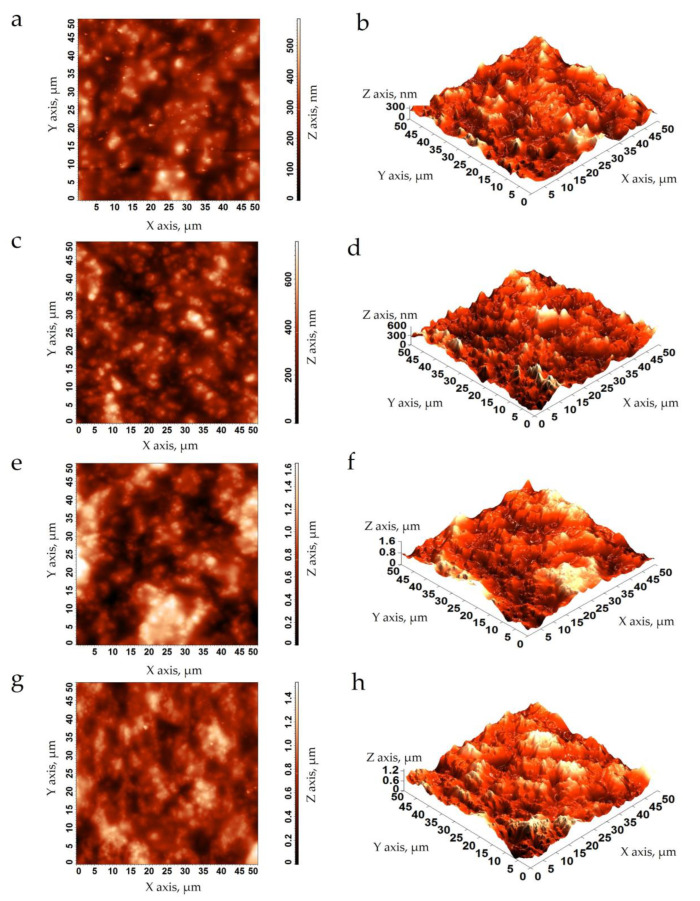
Atomic force micrographs of cassava starch films containing quinoa starch nanocrystals at (*w*/*w*): (**a**,**b**) 0%; (**c**,**d**) 2.5%; (**e**,**f**) 5.0% and (**g**,**h**) 7.5%. Left: 2D, and right: 3D micrographs.

It can be observed that the QSNC modified the topographies in CS films (Figure 4). According to the 3D topographies (Figure 4b,d,f,h), all CS films presented an irregular topography, with valleys, troughs and peaks in nanoscales. The peaks increased as a consequence of the QSNC concentration, possibly due to some migration of these nanocrystals to the film surface during drying. The 2D micrographs (Figure 4a,c,e,g) corroborated this interpretation. This behavior was also observed by scanning electron microscopy in starch films reinforced with SNCs from cassava [24], waxy maize [18] and taro [19]. In the 3D micrographs, the highest peak heights increased from ~300 to ~1200 nm with the increase of the QSNC concentrations.

As a consequence of the above-described behavior, the film’s average roughness (R_a_) increased from 85–102 to 205–247 nm, and the root mean square roughness (R_q_) increased from 108 to 261–309 nm (Table 1) as a consequence of the QSNC concentration increasing, possibly because some aggregates formed between QSNCs and amylose chains during film drying. The R_a_ and R_q_ values for the cassava starch films, with 0 and 2.5% QSNC concentrations, were similar to those observed for the cassava starch films and waxy maize starch films [43]. Valencia et al. [6] also determined the R_a_ and R_q_ values increased from 111 and 133 nm to 161 and 200 nm, respectively, when the concentration of Laponite© increased from 0 to 6% in the CS nanocomposite films. In a similar way, Dai et al. [25] observed that as the content of the cross-linked waxy maize SNC (0–12%) increased, the roughness of the cassava starch films increased from 1 to 12 nm.

#### 3.2.5. Film Gloss

Considering the gloss values determined at 60° (Table 1), all films can be classified as having low gloss surfaces (<70 units), according to Villalobos et al. [44]. Since gloss is a consequence of surface polishing that affects surface light diffraction [44], film gloss must vary because of the QSNC concentration effect on film roughness, as explained in Section 3.2.4. Indeed, the gloss of the air-side surface of films containing QSNCs reduced linearly as a function of both R_a_ (Equation (3), R^2^ = 0.82) and R_q_ (Equation (4), R^2^ = 0.86), with almost the same tendency (similar slopes). This was in accordance with the observations for other nanoparticles, such as Laponite© [6] and cellulose nanofibers [42] in starch films.

Gloss = −0.22 R_a_ + 67.9 (3)

Gloss = −0.18 R_q_ + 68.1 (4)

#### 3.2.6. Film Water Contact Angle

**Figure 5:**
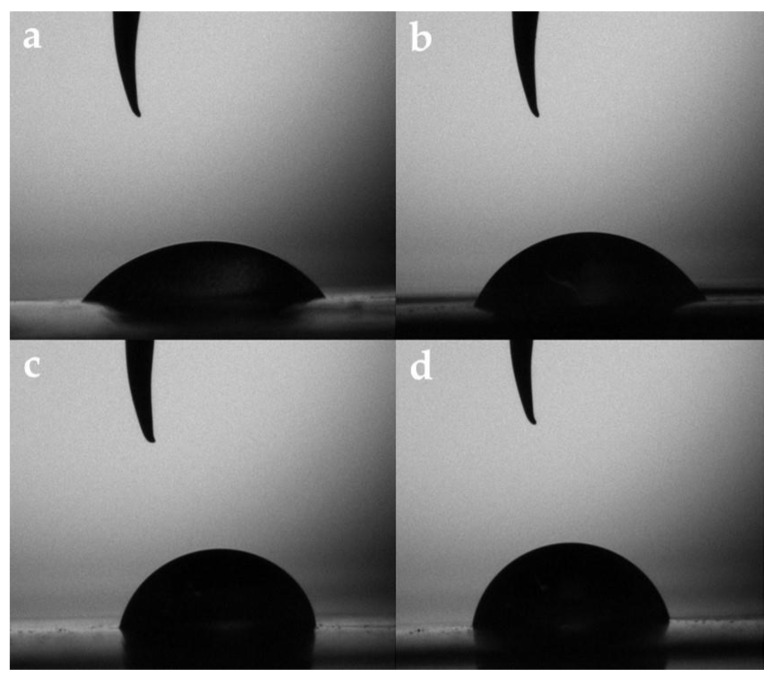
Water droplet images captured after 30 s of cassava starch films with quinoa starch nanocrystals at (*w*/*w*): (**a**) 0%; (**b**) 2.5%; (**c**) 5.0% and (**d**) 7.5%.

The increase in the QSNC concentration raised the film surface hydrophobicity; therefore, the droplets were smaller, had a larger base for the lower QSNC concentrations and were higher with a shorter base for the higher QSNC concentrations (Figure 5). The films reinforced at 0 and 2.5% presented a WCA lower than 65°, classifying them as hydrophilic surfaces, while those reinforced with higher concentrations presented values above 65° (Table 1) corresponded to those that were hydrophobic [6]. Nevertheless, this behavior of the WCA could also be associated with the effect of the QSNC concentration on R_a_ (Equation (5), R^2^ = 0.88) and R_q_ (Equation (6), R^2^ = 0.89).

WCA = 0.16 R_a_ + 38.6 (5)

WCA = 0.13 R_q_ + 38.4 (6)

The film-specific surface increased as a consequence of its roughness, contributing to raising the adhesion force between the water droplet and the film surface. Condés et al. [36] and Dai et al. [25], working on amaranth protein films reinforced with waxy maize SNC (0–12%) and cassava starch film containing cross-linked waxy maize SNC (0–12%), respectively, observed that the hydrophilicity of the films gradually decreased with the addition of SNC. This was attributed to SNC-matrix interactions (hydrogen bonds), which reduced the concentration of the hydrophilic groups on the film surface and their interaction with water.

#### 3.2.7. Film X-ray Diffraction Patterns

Film X-ray diffractograms were characterized by a wide peak around 20° (Figure 2b), which has been associated with the presence of amorphous material in the film [6,22] that resulted from the disruption of the CS granule crystalline regions during gelatinization. The presence of QSNCs at concentrations from 2.5 to 7.5% in the films caused the appearance of A-type crystalline pattern peaks (15°, 17°, and ~23°) and a slight increase in the intensity of the central peak, confirming QSNC presence and preservation of their crystallinity after film processing. This result was consistent with other works, which reported that the addition of SNCs in starch films resulted in the appearance of characteristic crystalline pattern peaks from the SNC source and that this intensity varied with their concentration [2,19,25]. It is important to mention that the estimation of the crystallinity index of the films was not possible because the studied concentrations of QSNCs did not significantly change the crystalline portion of the films since the diffraction patterns are similar to pure film and the nanoparticle appeared as traces in an amorphous material.

#### 3.2.8. Film Mechanical Properties

The CS film tensile strength (TS) and Young’s modulus (M) increased around 2.5 times when the QSNC concentration increased from 0 to 5% (Table 2), and then it reduced above this, i.e., for 7.5% of QSNC. Notably, the elongation at break (EB) decreased as a function of the QSNC concentration (Table 2). The TS and M behavior, which had an apparent maximum value at 5% of QSNC (Table 2), was probably due to the good exfoliation of nanoparticles inside the starch matrix at a low concentration and up to 5%, which allowed strong interfacial interactions between them. Thus, an effective transfer of stress between both fractions must have occurred [2,17,19].

Nevertheless, when the QSNC concentration increased to 7.5%, the exfoliation quality was lost due to the eventual SNC agglomeration that caused a loss of stress transfer effectiveness, as observed by Dai et al. [25] working on cassava starch films containing cross-linked waxy maize SNC, at a 12% concentration. Possibly, at levels above 5% of QSNCs, a higher self-aggregation of QSNCs occurred that reduced their specific surface areas, avoiding possible interactions with the CS matrix and leading to a micro-phase separation [8,18]. Therefore, the formation of QSNC-rich phases at higher concentrations could explain the breaking of the film at a 10% concentration during drying. This behavior has been observed by other authors, who found maximum TS and M values for different SNC concentrations in films: 2.5% [19] and 5% [10,18], for instance.

#### 3.2.9. Film Thermal Properties

**Figure 6:**
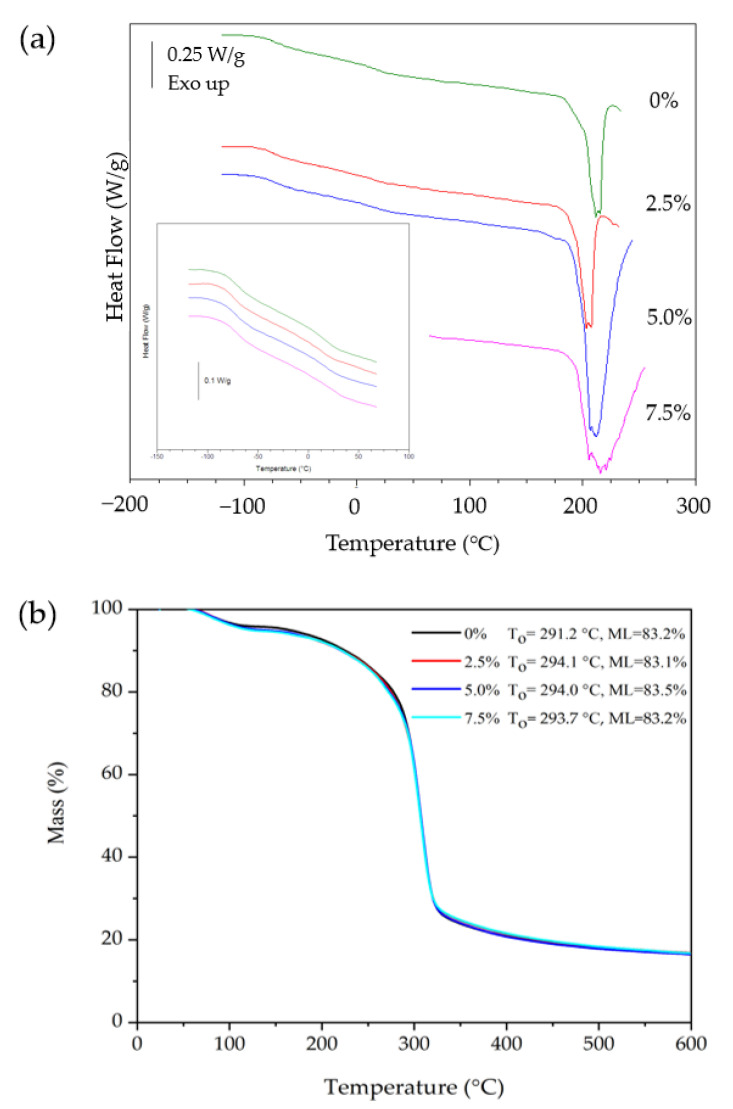
(**a**) Differential scanning calorimetric curves and (**b**) thermogravimetric curves of cassava starch films with quinoa starch nanocrystals at different concentrations. T_0_ = onset decomposition temperature, ML = mass loss.

The film thermal curves, determined by DSC (Figure 6a), evidenced two glass transition temperatures. The first T_gG_ (~−71.4 °C) was not affected by the QSNCs and was attributed to a glycerol-rich phase, while the second one, T_gS_, associated with the biopolymer-rich phase [45], increased from 15.0 ± 2.1 to 23.1 ± 3.5 °C when the QSNC concentration increased from 0 to 7.5% (Table 3). These results corroborate the increase in the film’s mechanical properties (Table 2) due to the presence of QSNCs. This behavior was due to an amylopectin chain molecular mobility restriction because of strong interactions with the reinforcing filler, probably by hydrogen bonding, of starch chains by QSNCs [16,19]. Similar findings were observed in the waxy maize starch films reinforced with waxy maize SNC (0–15%) and plasticized with sorbitol [16].

The film melting temperature (T_m_), which is linked to starch degradation, was not affected by the QSNC addition, remaining at around 213 °C (Table 3). This behavior was also observed in the starch films containing potato starch nanoparticles at similar concentrations (3 and 6%) [9]. Nevertheless, Li et al. [18], Martins et al. [2] and Mukurumbira et al. [19] observed a different behavior. They verified that T_m_ increased in starch films reinforced with waxy maize SNC (5%), potato SNC (0.1–0.3%) and taro SNC (2.5−10%) due to SNC-film matrix interactions, which increased film crystallinity. Thus, the behavior observed in this study could be related to QSNCs’ characteristic physical properties, particularly regarding their low crystallinity (~35%), which in the studied concentrations, did not influence T_m_. In contrast, the QSNC addition did increase film enthalpy from 56–61 to 161–147 J/g when the concentration increased from 0–2.5 to 5–7.5% (Table 3). This property can present different behaviors depending on the SNC concentration, its source and its subsequent interactions with the starch matrix [2,18,19].

According to the thermogravimetric film curves for the CS films containing 0–7.5% of QSNC (Figure 6b), the thermal degradation of the films occurred in two stages. The first stage, from 50 to 150 °C, corresponded to water loss, a characteristic phenomenon for a polysaccharide system of hydrophilic nature, while the second one, between 200 and 370 °C, was attributed to QSNC and other film component decompositions. The QSNC addition to the films did not affect the onset decomposition temperature of these materials, which remained at around 203 °C, or the overall mass loss, which remained at around 83% (Figure 6b). This behavior differed from that reported by Li et al. [18] and Piyada et al. [17], who observed that concentrations of 1–5% waxy maize SNC and 0–30% rice SNC, respectively, improved the thermal stability of starch films; however, this result was in accordance with those obtained by DSC, suggesting that QSNCs in the studied concentration range did not improve the film’s thermal stability.

#### 3.2.10. Fourier Transform Infrared Spectroscopy (FTIR)

**Figure 7:**
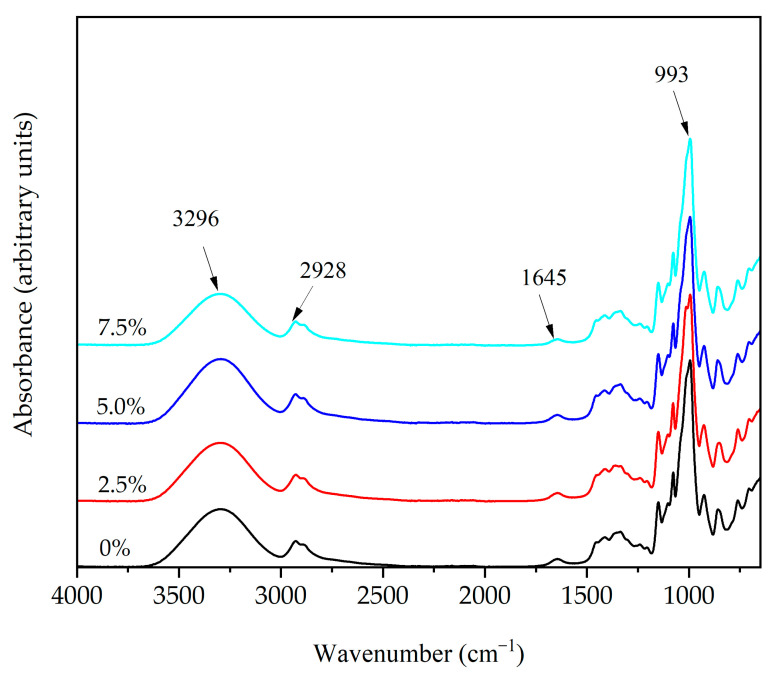
Fourier transform infrared spectra of cassava starch films reinforced with quinoa starch nanocrystals at different concentrations.

Film FTIR spectra (Figure 7) showed the main characteristic absorption bands attributed to starch at 3290 cm^−1^ (O-H), 2928 cm^−1^ (C-H), 1645 cm^−1^ (O-H), 1015 cm^−1^ (C-O-C), and 993 cm^−1^ (C-O) [46]. These bands did not shift with the QSNC addition (0–7.5%), probably due to chemical compatibility, or in other words, because these nanoparticles have the same chemical structure as CS (Figure 7) due to the functional groups remaining after the acid hydrolysis [14]. Nevertheless, the intensity of the band located at 3600–3000 cm^−1^ slightly decreased after the QSNC addition, possibly related to a diminution of the available hydroxyl groups due to intermolecular hydrogen bonding among the QSNC hydroxyl groups and the starch matrix [47]. Moreover, a lower intensity was identified in the region 950–1100 cm^−1^, which is broadly associated with ordered structures [48]. This could therefore indicate an intercalation of the structure of QSNC and the polymer chains or a difference in the structural change [49].

## 4. Conclusions

The physical and structural properties of the cassava starch films showed that the QSNC application as a reinforcing filler was viable and relevant, mainly in relation to the mechanical (tensile strength and Young’s modulus) and barrier (WVP) properties of the material without degradation of its other physical properties. In this study, 5% *w*/*w* of QSNC was the best concentration regarding the tensile strength and Young’s modulus. These properties were lower for the QSNC concentrations above or below 5%. At 5% of QSNC, the tensile strength increased up to 154% in relation to the films without nanoparticles. Moreover, CS films with 5% of QSNC presented a 17% lower WVP. The reduction of the tensile strength and Young’s modulus above 5% of QSNC was due to a loss in the nanoparticle dispersion quality.

The QSNC concentration also affected the film surface roughness, gloss, opacity and water contact angle. The effect of QSNCs on gloss and the water contact angle was due to the alteration caused by nanoparticles on air-side film roughness. The FTIR analysis suggested the formation of hydrogen bonding without forming any chemical bonding between QSNCs and cassava starch as a function of the QSNC concentration, which was indicated by its solubility in water and thermal properties. This work evidenced that QSNCs acted as a reinforcement of CS films, indicating their potential for the development of nanocomposite starch-based films, which can be used in food packaging applications.

## Figures and Tables

**Table 1:** Thickness, moisture content, solubility in water, water vapor permeability (WVP), color parameters, opacity, average roughness (R_a_), root mean square roughness (R_q_), gloss and water contact angle of cassava starch films containing quinoa starch nanocrystals (QSNC) *.

	QSNC Concentration (%)			
Properties	0	2.5	5.0	7.5
Thickness (mm)	0.103 ± 0.002 ^a^	0.102 ± 0.003 ^a^	0.100 ± 0.003 ^a^	0.106 ± 0.002 ^a^
Moisture content (%)	11.8 ± 0.4 ^a^	11.0 ± 0.6 ^a^	10.5 ± 0.3 ^a^	10.7 ± 0.4 ^a^
Solubility in water (%)	25.1 ± 1.4 ^a^	24.3 ± 0.7 ^a^	22.9 ± 1.0 ^a^	23.9 ± 0.7 ^a^
WVP (g.mm/m^2^·h·kPa)	0.54 ± 0.01 ^a^	0.50 ± 0.01 ^a,b^	0.45 ± 0.03 ^b^	0.52 ± 0.03 ^a,b^
L*	91.3 ± 0.0 ^b^	91.3 ± 0.2 ^b^	91.7 ± 0.1 ^b^	92.2 ± 0.0 ^a^
a*	−0.67 ± 0.01 ^a,b^	−0.67 ± 0.01 ^b^	−0.66 ± 0.01 ^b^	−0.69 ± 0.01 ^a^
b*	2.4 ± 0.0 ^b^	2.5 ± 0.0 ^b^	2.5 ± 0.1 ^b^	2.8 ± 0.1 ^a^
∆E*	3.2 ± 0.0 ^a^	3.1 ±0.2 ^a^	2.9 ±0.1 ^a^	2.4 ± 0.1 ^b^
Opacity	0.51 ± 0.08 ^d^	1.19 ± 0.18 ^c^	2.05 ± 0.09 ^b^	3.29 ± 0.08 ^a^
R_a_ (nm)	85 ± 1 ^b^	102 ± 6 ^b^	205 ± 8 ^a^	247 ± 33 ^a^
R_q_ (nm)	108 ± 2 ^c^	129 ± 7 ^b^	261 ± 17 ^a^	309 ± 39 ^a^
Gloss (GU)	58.3 ± 5.0 ^a^	35.2 ± 2.6 ^b^	19.5 ± 0.8 ^c^	15.6 ± 0.3 ^c^
Contact angle (°)	47.9 ± 2.3 ^b^	57.8 ± 2.8 ^b^	75.3 ± 2.8 ^a^	73.5 ± 1.4 ^a^

* Mean values ± standard deviation. Values in the same row with different small letters differ significantly by Tukey’s test (*p* ≤ 0.05).

**Table 2:** Cassava film mechanical properties with quinoa starch nanocrystals (QSNC) at different concentrations *.

QSNC (%)	TS (MPa)	EB (%)	M (MPa/%)
0	6.5 ± 0.5 ^c^	10.2 ± 1.0 ^a^	2.8 ± 0.2 ^c^
2.5	8.4 ± 0.5 ^b,c^	7.4 ± 0.9 ^b^	3.7 ± 0.2 ^b,c^
5.0	16.5 ± 2.5 ^a^	8.7 ± 0.6 ^a,b^	6.6 ± 0.8 ^a^
7.5	11.8 ± 0.3 ^b^	7.1 ± 0.7 ^b^	4.8 ± 0.1 ^b^

* Mean values ± standard deviation. Values in the same column with different letters differ significantly by Tukey’s test (*p* ≤ 0.05). TS, tensile strength; EB, elongation at break; M, elastic module.

**Table 3:** Cassava film thermal properties with quinoa starch nanocrystals (QSNC) at different concentrations *.

QSNC (%)	T_gG_ (°C)	T_gS_ (°C)	T_m_ (°C)	∆H_m_ (J/g)
0	−70.1 ± 3.0 ^a^	15.0 ± 2.1 ^b^	212.8 ± 2.3 ^a^	56.2 ± 7.0 ^b^
2.5	−71.7 ± 2.1 ^a^	13.6 ± 1.4 ^b^	208.1 ± 5.5 ^a^	60.9 ± 3.7 ^b^
5.0	−71.6 ± 1.1 ^a^	17.7 ± 0.9 ^a,b^	214.5 ± 2.4 ^a^	161.2 ± 14.6 ^a^
7.5	−72.2 ± 2.1 ^a^	23.1 ± 3.5 ^a^	215.8 ± 2.5 ^a^	147.2 ± 5.9 ^a^

* Mean values ± standard deviation. Values in the same column with different small letters differ significantly by Tukey’s test (*p* ≤ 0.05). T_gG_ and T_gS_, glass transition temperatures associated with glycerol and starch-rich fractions, respectively; T_m_, melting temperature; ∆H_m_, enthalpy of melting.

## Data Availability

The datasets generated during and/or analyzed during the current study are available from the corresponding author upon reasonable request.
